# Supplementary material for: A novel epitope tagging system to visualize and monitor antigens in live cells with chromobodies
Source: Sci Rep. 2020 Aug 31;10:14267. doi: 10.1038/s41598-020-71091-x (PMC7459311; doi:10.1038/s41598-020-71091-x)
Supplement: Supplementary file 1 — Supplementary information. [file 41598_2020_71091_MOESM1_ESM.pdf]

A novel epitope tagging system to visualize and monitor antigens in live cells with chromobodies

Bjoern Traenkle[1,2*], *Sören Segan*[2,*], *Funmilayo O. Fagbadebo*[1], *Philipp D. Kaiser*[2] *and Ulrich Rothbauer*[1,2]

[1] Pharmaceutical Biotechnology, Eberhard Karls University Tuebingen, Germany

[2] Natural and Medical Sciences Institute at the University of Tuebingen, Germany

[*] contributed equally

Correspondence:

Prof. Dr. Ulrich Rothbauer, Natural and Medical Sciences Institute at the University of Tuebingen

Markwiesenstr. 55, 72770 Reutlingen, Germany.

E-mail: ulrich.rothbauer@uni-tuebingen.de

Phone: +49 7121 51530-415

Fax: +49 7121 51530-816

17 Supplementary Information

oligonuclotide name	sequence (5‘ – 3‘)
PepTag-ACTB-for	ATAGGTACCTGAAAGATCAGCAGCTGCTGGGCATTTGGGGAGGCAGCGAT<br>GATGATATCGCCGCGCTCGTC
PepTag-ACTB-rev	TATGGTACCTTTCCACCGCCATGGTGGCGGTGGCGACCGGTAGCGCTAG
AAVS1-HA-L-for	CCTCTCTAGTCTGTGCTAGCTC
AAVS1-HA-R-rev	GAAGGAGGAGGCCTAAGGATGG
AAVS1-vor-HA-L-for	CGGAACTCTGCCCTCTAACG
AAVS1-T2A-rev	GGGATTCTCCTCCACGTCAC
PCNA-PepTag-for	CTGAAGGACCAGCAGCTCCTCGGCATCTGGTAGTCTAGAGTCGAGATC
PCNA-PepTag-rev	GTACCTCTCCACGGCGGATCCCCCGCCTCCAGATCCTTCTTCATCCTC
PepNB-DNA-fragment	AGATCTCCGGCCATGGCTGACGTGCAGCTGCAGGAGAGCGGCGGCGGCCT<br>GGTGCAGCCCGGCGGCAGCCTGAGGCTGAGCTGCGCCGCCAGCGGCAAC<br>ATCGTGAGCATCGACGCCGCCGGCTGGTTCAGGCAGGCCCCCGGCAAGCA<br>GAGGGAGCCCGTGGCCACCATCCTGACCGGCGGCGCCACCAACTACGCC<br>GACAGCGTGAAGGGCAGGTTCACCATCAGCAGGGACAACGCCAAGAACAC<br>CGTGTACCTGCAGATGAACAGCCTGAAGCCCGAGGACACCGCCGTGTACTA<br>CTGCTACGCCCCCATGATCTACTACGGCGGCAGGTACAGCGACTACTGGG<br>GCCAGGGCACCCAGGTCACC
Ub-R-PepCB	ATGCAGATCTTCGTGAAGACTCTGACTGGTAAGACCATCACCCTCGA<br>GGTTGAGCCCAGTGACACCATTGAGAATGTCAAGGCAAAGATCCAA<br>GATAAGGAAGGCATCCCTCCTGACCAGCAGAGGCTGATCTTTGCTG<br>GAAAACAGCTGGAAGATGGGCGCACCCTGTCTGACTACAACATCCA<br>GAAAGAGTCCACCCTGCACCTGGTACTCCGTCTCAGAGGTGGGAGG<br>GCTCAGGTGCAGCTGCAGGAGAGCGGCGGCGGCCTGGTGCAGCCC<br>GGCGGCAGCCTGAGGCTGAGCTGCGCCGCCAGCGGCAACATCGTG<br>AGCATCGACGCCGCCGGCTGGTTCAGGCAGGCCCCCGGCAAGCAG<br>AGGGAGCCCGTGGCCACCATCCTGACCGGCGGCGCCACCAACTAC<br>GCCGACAGCGTGAAGGGCAGGTTCACCATCAGCAGGGACAACGCC<br>AAGAACACCGTGTACCTCCAGATGAACAGCCTGAAGCCCGAGGACA<br>CCGCCGTGTACTACTGCTACGCCCCCATGATCTACTACGGCGGCAG<br>GTACAGCGACTACTGGGGCCAGGGCACCCAGGTCACCGTCTCCTCC<br>GGAGGCGGGGGAAGCGGAGGCGGGGGAAGCGGAGGCGGGGGAT<br>CCATGGTGAGCAAGGGCGAAGAGCTGATTAAGGAGAACATGCACAT<br>GAAGCTGTACATGGAGGGCACCGTGAACAACCACCACTTCAAGTGC<br>ACATCCGAGGGCGAAGGCAAGCCCTACGAGGGCACCCAGACCATG<br>AGAATCAAGGTGGTCGAGGGCGGCCCTCTCCCCTTCGCCTTCGACA<br>TCCTGGCTACCAGCTTCATGTACGGCAGCAGAACCTTCATCAACCAC<br>ACCCAGGGCATCCCCGACTTCTTTAAGCAGTCCTTCCCTGAGGGCTT<br>CACATGGGAGAGAGTCACCACATACGAAGACGGGGGCGTGCTGAC<br>CGCTACCCAGGACACCAGCCTCCAGGACGGCTGCCTCATCTACAAC<br>GTCAAGATCAGAGGGGTGAACTTCCCATCCAACGGCCCTGTGATGC<br>AGAAGAAAACACTCGGCTGGGAGGCCAACACCGAGATGCTGTACCC<br>CGCTGACGGCGGCCTGGAAGGCAGAAGCGACATGGCCCTGAAGCT<br>CGTGGGCGGGGGCCACCTGATCTGCAACTTCAAGACCACATACAGA<br>TCCAAGAAACCCGCTAAGAACCTCAAGATGCCCGGCGTCTACTATGT<br>GGACCACAGACTGGAAAGAATCAAGGAGGCCGACAAAGAGACCTAC<br>GTCGAGCAGCACGAGGTGGCTGTGGCCAGATACTGCGACCTCCCTA<br>GCAAACTGGGGCACAAACTTAATTGA
vimentin-PepTag-for	GAGGTACCTGAAGGACCAGCAGCTCCTCGGCATCTGGTGAGATCCACCGG<br>ATCTAGATAACTGAT

vimentin-PepTag-rev	CAGGTACCTCTCCACGGCGGATCCCCCGCCTCCTGGTTCAAGGTCATCGTGATG
PepTag-eGFP-for	AAAGGTACCTGAAAGATCAGCAGCTGCTGGGCATTTGGGGAGGCAGCGTGAGCAAGGGCGAGGAGC
PepTag-eGFP-rev	TTTCAGGTACCTTTCCACCGCCATGCTAGCGGATCTGACGGTTC
eGFP-NLS-for	AGAAGAGGAAGGTTTGATAAAGCGGCCGCGACTCT
eGFP-NLS-rev	TCTTAGGGCTGCCTCCCTTGTACAGCTCGTCCATGCC
Del-GFP-fwd	GGCGAAGGGCAAGGGCAA
Del-GFP-rev	GGTGGCGACCGGTACCAGTAC
Del-mCherry-for	TCCACCAGGTCCGTGTCC
Del-mCherry-rev	CATGGTGGCGACCGGTAG
BkB-pepAct-for	CATTATTGAAACAGCGATGAGGATCCACCGGATCTAGATAAC
Bkb-pepAct-rev	CCAAATGCCCAGCAGCTG
mutMiro1-for	TGGAAGTGGTGGCGGAGGTAGCATGAAGAAAGACGTGCGG
mutMiro1-rev	CCGCCTCCAGAACCTCCTCCACCGCTGCTCCAATGGCTCAC
Miro1-for	AGCAGCTGCTGGGCATTTGGGGTGGAGGAGGTTCTGGAG
Miro1-rev	TCATCGCTGTTTCAATAATGCTTTG

Table S1: List of DNA oligonucleotides and synthesized gene fragments used in this study

	**Expression constructs/stable cell lines**
1.	U2OS_E02 (this study)
2.	BHK cells containing multiple lac-operon repeats [1]
3.	PepCB (tagRFP) (this study)
4.	PepCB (eGFP) (this study)
5.	lamin-CB [2]
6.	PCNA-CB-TagRFP [3]
7.	PepNb-Myc-KKK-His$_6$ (this study)
8.	PepNb-Sort-His$_6$ (this study)
9.	AAVS1_EF1-α-Ub-R-ACT-CB [4]
10.	AAVS1_EF1-α-Ub-R-PepCB (this study)
11.	mCherry-vimentin [5]
12.	mCherry-vimentin$_{Pep}$ (this study)
13.	BC2T-eGFP [6]
14.	${}_{Pep}$GFP (this study)
15.	${}_{Pep}$actin (this study)
16.	BC2T-actin [6]
17.	${}_{Pep}$GFP-tubulin (this study)
18.	PamCherry-tubulin [7]
19.	GFP-PCNA$_{Pep}$ (this study)
20.	GFP-PCNA [8]
21.	eGFP-NLS (this study)
22.	${}_{Pep}$GFP-NLS (this study)
23.	lacI-GBP [9]
24.	Lamin-GBP [9]
25.	GFP-actin (Takara Bio USA, Inc.)
26.	actin-CB [3]
27.	eGFP (Takara Bio USA, Inc.)
28.	${}_{Pep}$Miro1 (this study)
29.	Miro1$_{His6}$ (kindly provided by Julia Fitzgerald)
30.	PCNA$_{Pep}$ (this study)
31.	VIM$_{Pep}$ (this study)

Table S2: List of expression constructs and stable cell lines used in this study

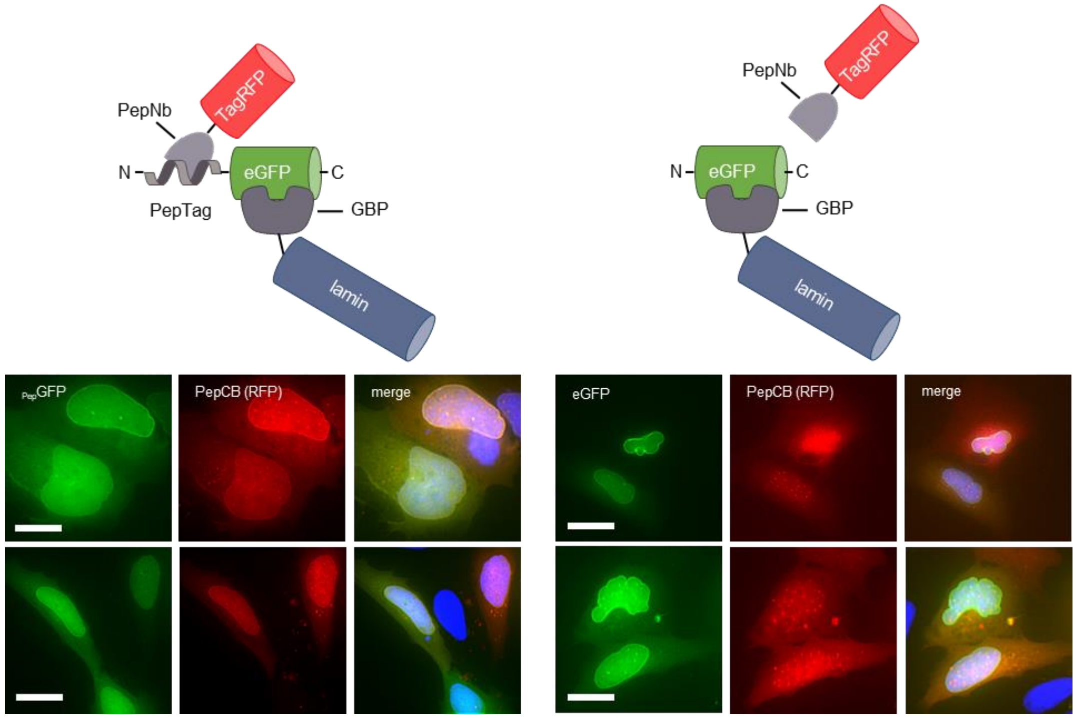

Supplementary Figure 1: Nuclear lamina-based interaction assay of the PepCB and $_{Pep}$GFP *in cellulo*.

Top panels illustrate assay principle: nuclear lamina-resident GBP-lamin B1 recruits co-expressed $_{Pep}$GFP along with PepCB (TagRFP); control situation depicted on the right. Representative fluorescence images of living U2OS cells transiently co-expressing PepCB, GBP-lamin B1, and $_{Pep}$GFP (lower left panel) or eGFP (lower right panel). Fluorescence colocalization at the nuclear lamina indicates PepCB binding to $_{Pep}$GFP. Scale bar 25 µm.

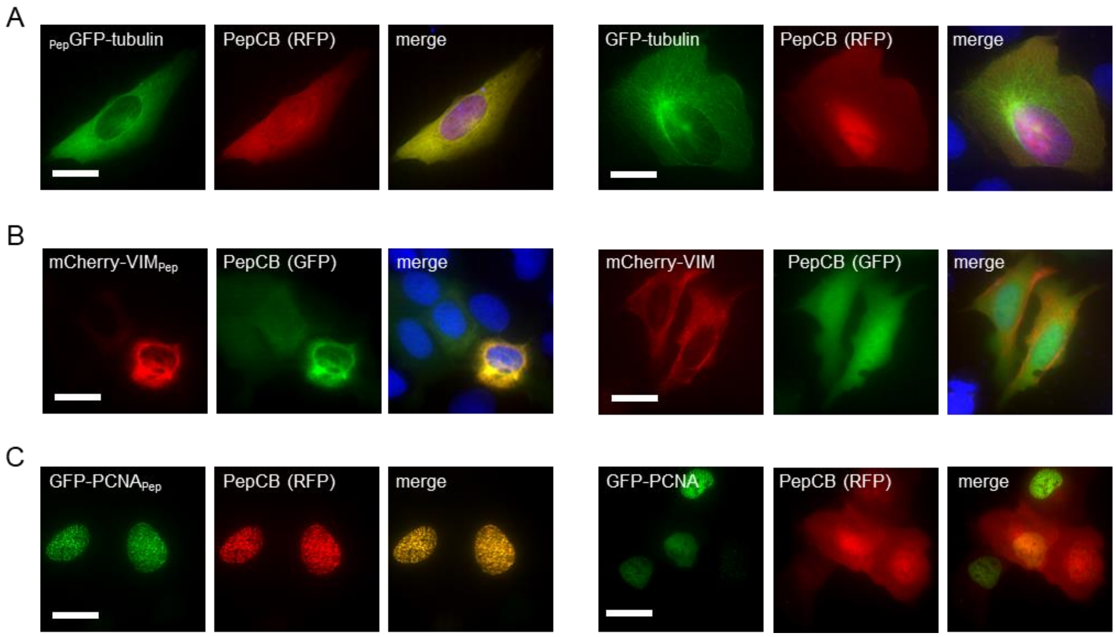

Supplementary Figure 2: PepCB visualizes Pep-tagged proteins in living cells.

PepCB with fluorescently tagged antigens: Representative fluorescence images of living U2OS cells transiently expressing PepCB labeled either with eGFP (GFP) or TagRFP (RFP) in combination with fluorescently tagged antigens: (**A**) ${}_{Pep}$GFP-tubulin, (**B**) mCherry-VIM$_{Pep}$, or (**C**) GFP-PCNA$_{Pep}$ (left panels) or the corresponding controls without PepTag (right panels).

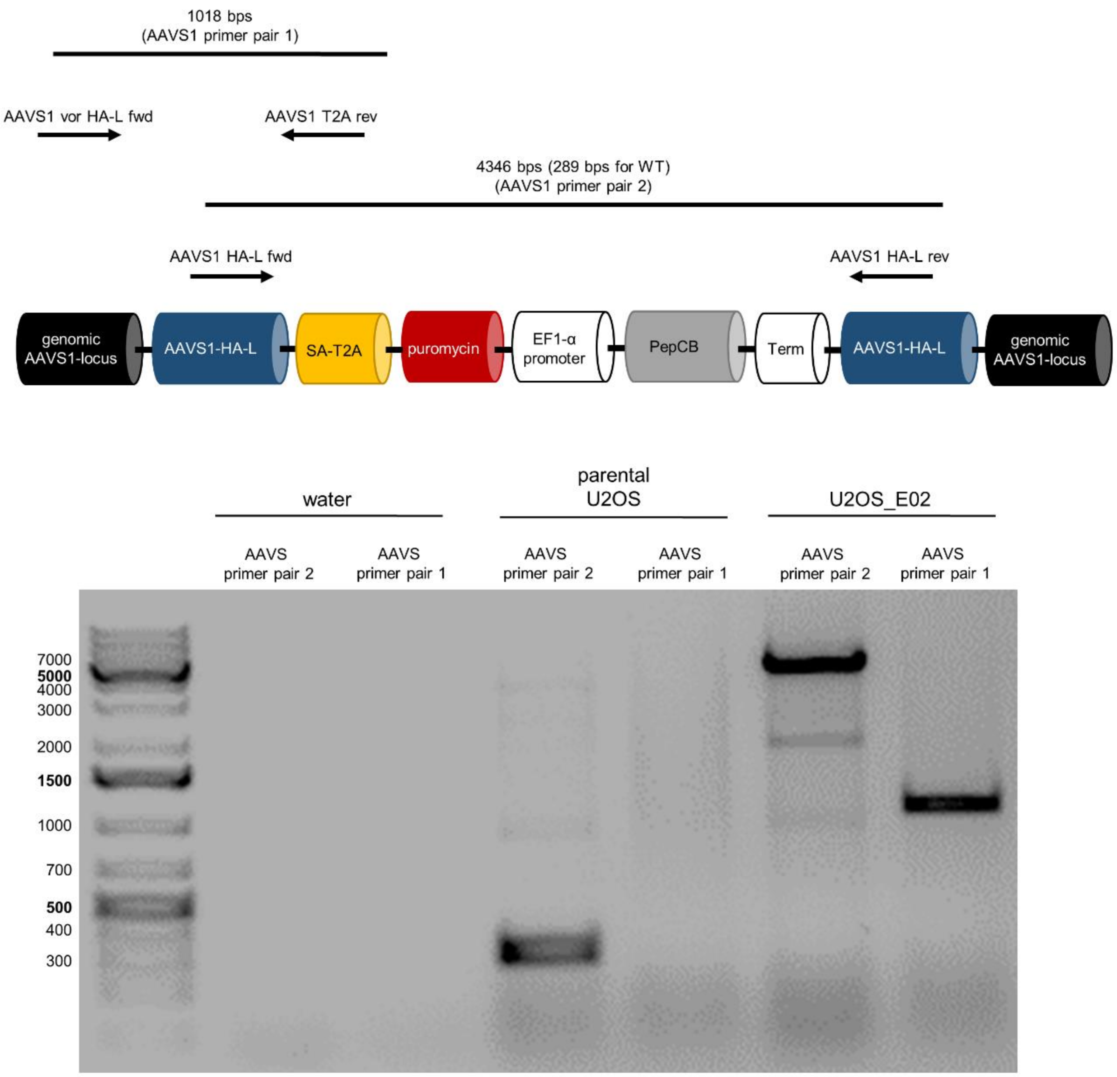

Supplementary Figure 3: PCR-based genotyping of CRISPR-engineered U2OS cell line. Schematic outline of genetic elements at the AAVS1 locus after integration of the PepCB. Arrows indicate the position of primers used to confirm correct genomic integration.

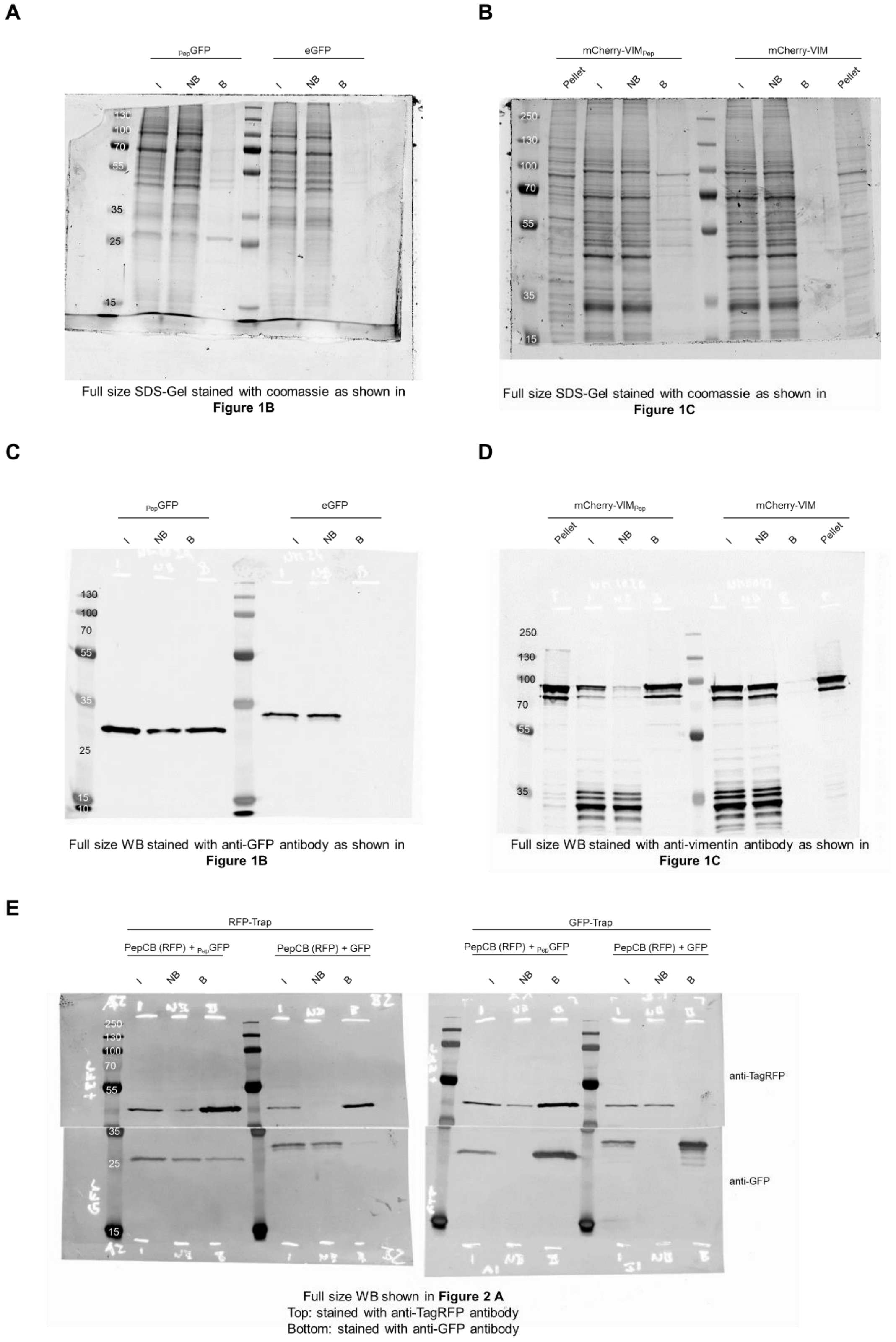

Supplementary Figure 4: Full size SDS-Gels stained with coomassie and Western Blots

(**A**) Full size SDS-Gel stained with coomassie as shown in **Fig.1 B,** (**B**) full size SDS-Gel stained with coomassie as shown in **Fig. 1 C;** (**C**) Full size Western blot stained with anti-GFP antibody as shown in **Fig. 1 B;** (**D**) full size WB stained with anti-vimentin antibody as shown in **Fig. 1 C;** (**E**) Full size WB shown in **Fig. 2 A** top: stained with anti-TagRFP antibody, bottom: stained with anti-GFP antibody.

Supplementary Video 1:

U2OS cells transiently co-expressing GFP-$PCNA_{Pep}$ and the red fluorescent PepCB. Time interval 1 h, scale bar 50 µm.

Supplementary Video 2:

U2OS cells transiently co-expressing $_{Pep}$actin and the red fluorescent PepCB. Cell were exposed to cytochalasin D for 10 min, followed by 30 min recovery. Time interval 5 min, scale bar 50 µm.

References

1 Tsukamoto, T. *et al.* Visualization of gene activity in living cells. *Nat Cell Biol* **2**, 871-878, doi:10.1038/35046510 (2000).

2 Zolghadr, K., Gregor, J., Leonhardt, H. & Rothbauer, U. Case study on live cell apoptosis-assay using lamin-chromobody cell-lines for high-content analysis. *Methods Mol Biol* **911**, 569-575, doi:10.1007/978-1-61779-968-6_36 (2012).

3 Panza, P., Maier, J., Schmees, C., Rothbauer, U. & Sollner, C. Live imaging of endogenous protein dynamics in zebrafish using chromobodies. *Development* **142**, 1879-1884, doi:10.1242/dev.118943 (2015).

4 Keller, B. M. *et al.* A Strategy to Optimize the Generation of Stable Chromobody Cell Lines for Visualization and Quantification of Endogenous Proteins in Living Cells. *Antibodies (Basel)* **8**, doi:10.3390/antib8010010. (2019).

5 Maier, J., Traenkle, B. & Rothbauer, U. Real-time analysis of epithelial-mesenchymal transition using fluorescent single-domain antibodies. *Scientific reports* **5**, 13402 (2015).

6 Braun, M. B. *et al.* Peptides in headlock–a novel high-affinity and versatile peptide-binding nanobody for proteomics and microscopy. *Scientific reports* **6** (2016).

7 Subach, F. V. *et al.* Photoactivatable mCherry for high-resolution two-color fluorescence microscopy. *Nature methods* **6**, 153-159 (2009).

8 Leonhardt, H. *et al.* Dynamics of DNA replication factories in living cells. *The Journal of cell biology* **149**, 271-280 (2000).

9 Zolghadr, K. *et al.* A Fluorescent Two-hybrid Assay for Direct Visualization of Protein Interactions in Living Cells. *Molecular & cellular proteomics : MCP* **7**, 2279-2287, doi:10.1074/mcp.M700548-MCP200 (2008).
