# Supplementary figures and images for: A novel epitope tagging system to visualize and monitor antigens in live cells with chromobodies
Source: Sci Rep. 2020 Aug 31;10:14267. doi: 10.1038/s41598-020-71091-x (PMC7459311; doi:10.1038/s41598-020-71091-x)

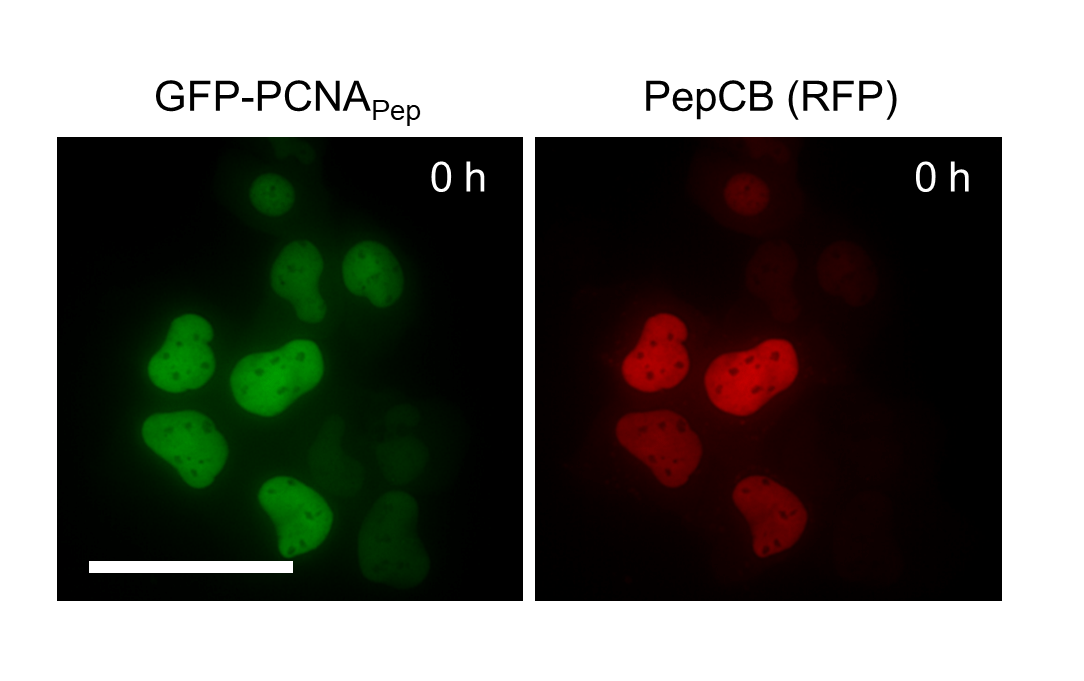

Supplement: Supplementary file 2 — Supplementary video 1. [file 41598_2020_71091_MOESM2_ESM.gif]

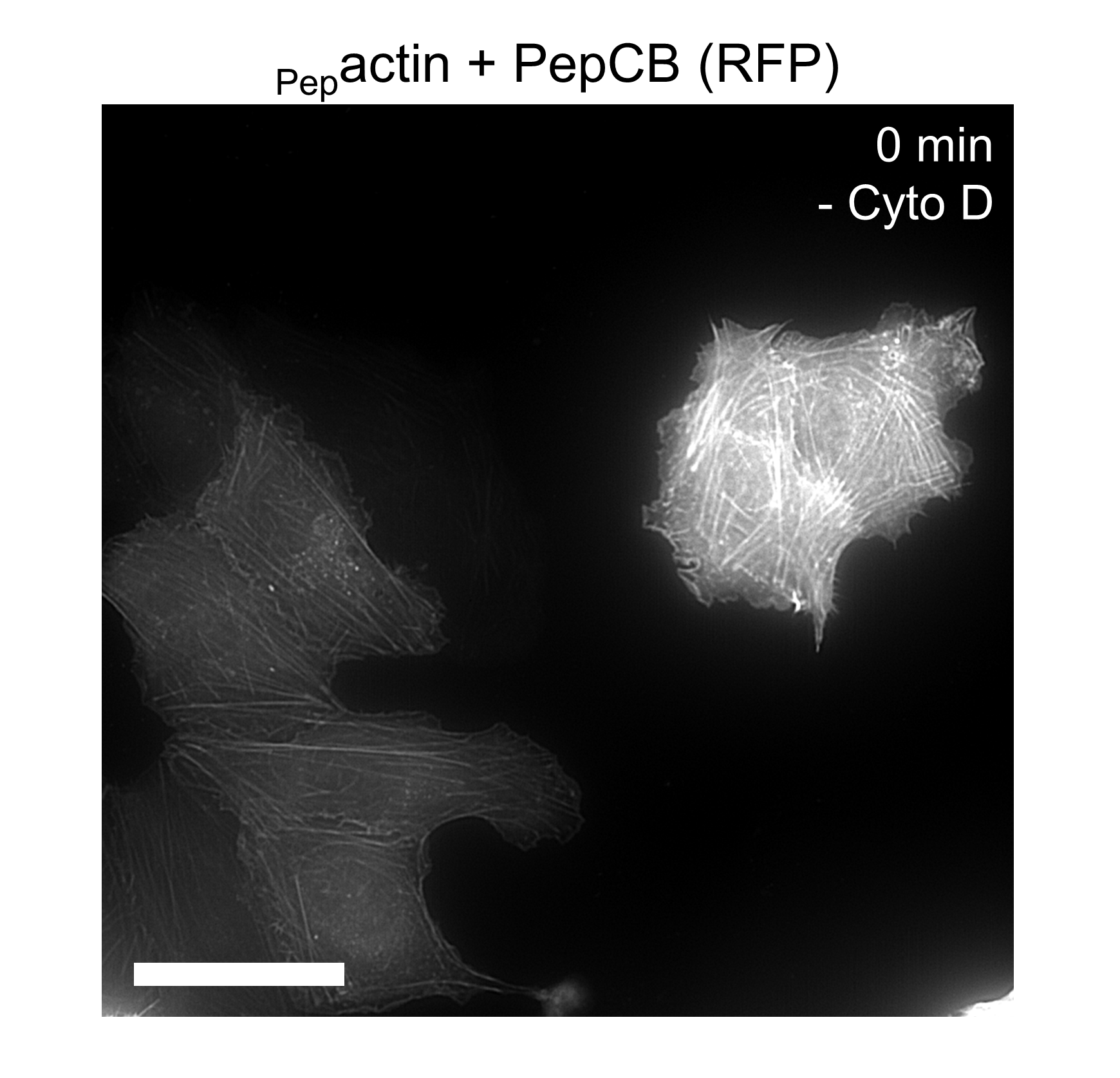

Supplement: Supplementary file 3 — Supplementary video 2. [file 41598_2020_71091_MOESM3_ESM.gif]
